# Various Rejuvenation Behaviors of Zr-Based Metallic Glass by Cryogenic Cycling Treatment with Different Casting Temperatures

**DOI:** 10.1186/s11671-018-2816-7

**Published:** 2018-12-06

**Authors:** Wei Guo, Rui Yamada, Junji Saida, Shulin Lü, Shusen Wu

**Affiliations:** 1 State Key Lab of Materials Processing and Die & Mould Technology, Huazhong University of Science and Technology, 1037 Luoyu Road, Wuhan, 430074 China; 2 Frontier Research Institute for Interdisciplinary Sciences, Tohoku University, Sendai, 980-8578 Japan

**Keywords:** Metallic glass, Rejuvenation, Cryogenic cycling, Casting temperature, Heterogeneity

## Abstract

The rejuvenation behavior of an Zr_50_Cu_40_Al_10_ (at.%) metallic glass upon cryogenic cycling treatment has been investigated. At a high casting temperature, the microstructure of the glass is quite homogenous and thus, internal stress cannot be generated during cycling. Therefore, the glass cannot be rejuvenated by cryogenic cycling treatment. In the contrary, by lowering the casting temperature, nano-sized heterogeneity can be induced and subsequently generates the internal stress and rejuvenates the glass. Once the glass is rejuvenated, the more induced free volume can plasticize the glass with a higher plastic strain. These findings point out that the synthesis conditions can tailor the heterogeneity of the glass and subsequently affect the following rejuvenation behavior upon thermal treatment. It can also help understand the mechanisms of rejuvenation of metallic glass upon cryogenic cycling treatment.

## Background

The bulk metallic glasses (BMGs) have attracted a lot of interests because of their superior mechanical properties such as high fracture strength and large elastic limit, which originates from their unique long-range disordered microstructures [1–3]. To suppress the nucleation and growth of crystalline phase during solidification, rapid quenching techniques are always required during the fabrication of BMGs [4–6]. The non-equilibrium solidification process makes BMGs possess higher configurational potential energy compared with their crystalline counterparts [7]. Thus, during annealing, the microstructures of BMGs tend to change toward a lower energy state (relaxation), which makes them more like the crystalline counterparts [8]. The so-called relaxation process of BMGs always degrades the properties of them, especially the mechanical properties, e.g., the embrittlement of BMGs after relaxation [9]. Furthermore, the BMGs can even crystallize by supplying thermal or mechanical energy. Dudina et al. have investigated the crystallization behavior of Ti-Cu metallic glass under high-current density electrical pulses [10]. They found that the crystallized microstructures of treated metallic glass vary with different pulse parameter and the crystalline phase can be as small as nano-size, which proves local melting and solidification during electrical pulse. In the contrary, the metastable BMGs can be also tailored to a higher energy state by both thermal and mechanical methods (rejuvenation), such as recovery annealing and severe plastic deformation [11–13]. Recently, Ketov et al. have found a novel deep cryogenic cycling treatment (DCT) to rejuvenate the BMGs, in which the samples are cooled and heated cyclically during room and cryogenic temperature (77 K) [14]. The mechanism for this rejuvenation is considered to be the intrinsic heterogeneous structure of amorphous phase, which generates internal stress during cooling and heating. In this study, by using our original developed DCT instrument, the rejuvenation behavior of Zr_50_Cu_40_Al_10_ (at.%) during DCT have been investigated with cycling number of 30, denoted as DCT30. Two kinds of casting temperatures have been chosen by varying the heating current during copper mold casting, i.e., 9 A (high temperature) and 7 A (low temperature), denoted as HT and LT, respectively. The microstructures and mechanical properties of each sample are investigated in detail.

## Methods

### Sample Preparation

Master alloys were prepared by arc-melting high-purity Cu, Zr, and Al metal pieces in a Ti-gettered argon atmosphere in a water-cooled copper hearth. The BMG was fabricated by casting the master alloy into a copper mold to produce a 2-mm-diameter rod-shaped sample (As-cast sample). The original instrument to conduct DCT has been described in our previous study [11]. By using this instrument, the samples can be cyclically cooled and heated between room temperature and 113 K.

### Sample Characterization

The structures of the samples were examined by X-ray diffraction (XRD; Bruker D8 Advance) with Cu Kα radiation, and transmission electron microscopy (TEM, JEOL JEM-2100F) with an acceleration voltage of 200 kV. The glass transition temperature (*T*_*g*_) and the onset crystalline temperature (*T*_*x*_) were measured by differential scanning calorimeter (DSC) in argon at a heating rate of 20 K/min. The specific heat capacities were measured by comparing them with a sapphire standard sample. The density was measured using an Ar gas pycnometer (AccuPyc II 1340, Micromeritics Co. Ltd.). Compression tests were performed at a strain rate of 5 × 10^−4^ s^−1^ at room temperature using an Instron 5982 mechanical testing machine. Multiple compression tests using at least four samples each were conducted to confirm the reproducibility.

## Results and Discussion

### Rejuvenation Behavior of HT Samples

**Fig. 1:**
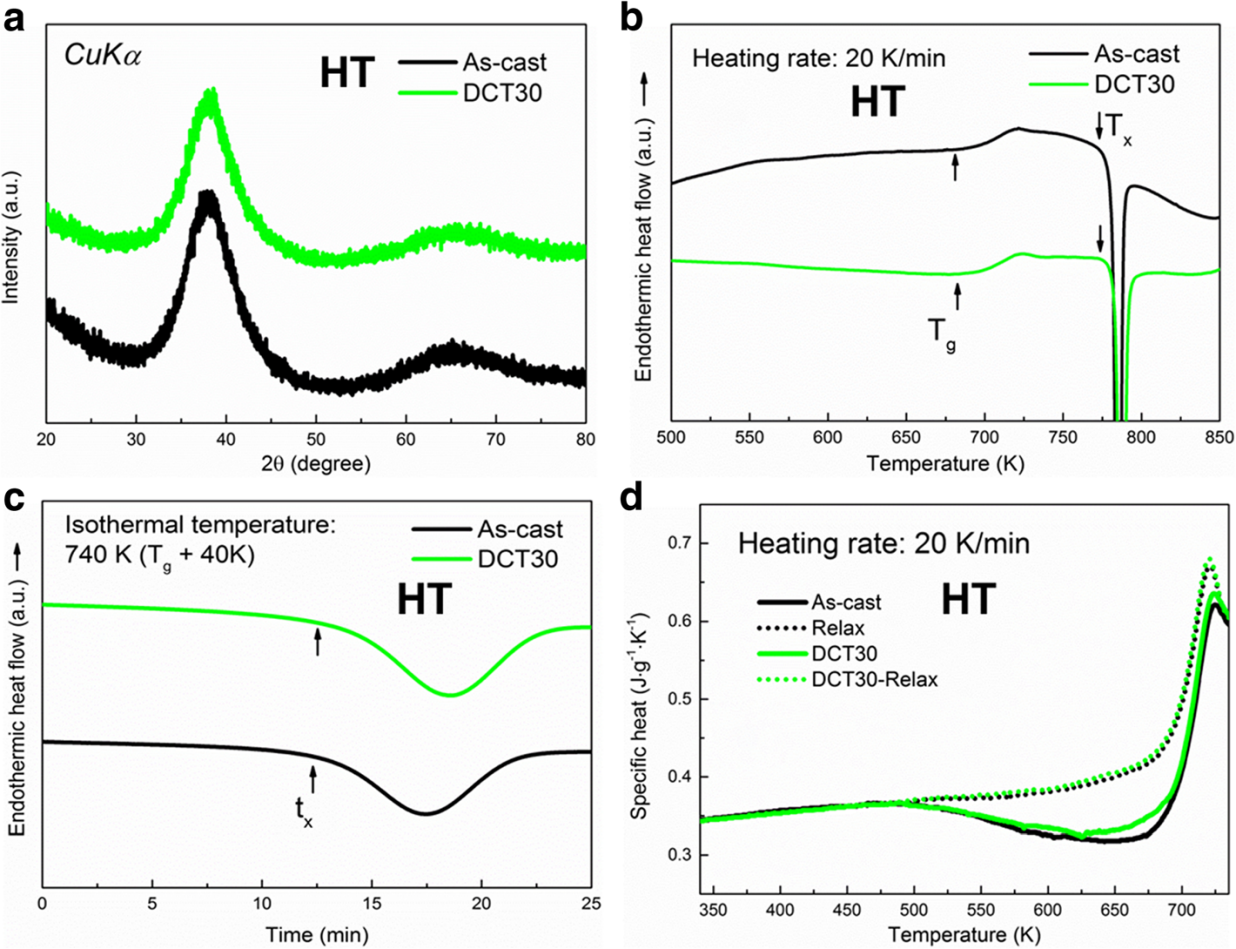
**a** XRD and **b** DSC curves of both As-cast and DCT30 samples cast at HT, **c** heat-flow as a function of time during isothermal annealing (740 K), and (**d**) specific heat of both As-cast and DCT30 samples cast at HT

Figure 1a shows the XRD patterns of both As-cast and DCT30 for HT samples, which exhibits similar board peak of amorphous phase without any obvious crystalline peaks. The DSC curves of both samples are shown in Fig. 1b, in which *T*_*g*_ and *T*_*x*_ are pointed out for each sample. Similar to XRD results, *T*_*g*_ and *T*_*x*_ for both samples are also very close, i.e., 690 K and 780 K for As-cast and 688 K and 781 K for DCT30, respectively. These results indicate that the amorphous phase does not have great changes during DCT, such as crystallization. Figure 1c shows the heat flow of both samples upon isothermal annealing at 740 K (1.07 *T*_*g*_), in which the incubation time of crystallization (*t*_*x*_) can be observed. By measuring the point of intersection before and during crystallization, *t*_*x*_ are found to be 12.6 and 12.5 min for As-cast and DCT30, respectively. The similar *t*_*x*_ also suggest that the resistance for both samples against crystallization are very alike. Furthermore, to evaluate the rejuvenation behavior more precisely, relaxation enthalpy (*ΔH*_relax_) are always used [14, 15], given as follows:

1 $$ \Delta  {H}_{relax}={\int}_{RT}^T\Delta  {C}_p dT, $$

where *ΔC*_*p*_ = *C*_*p,s*_ − *C*_*p,r*_, and *C*_*p,s*_ and *C*_*p,r*_ are the specific heats of the sample and its relaxed state, respectively. In the present study, the relaxed state was obtained by annealing at 725 K (~ 1.05 *T*_*g*_) for 2 min followed by a 20 K/min cooling. The specific heat curves of both samples and their relaxed state are plotted in Fig. 1d. Based on Eq. (1), *ΔH*_relax_ for As-cast and DCT30 was calculated to be ~ 12.6 J/g and 12.9 J/g, respectively. The similar *ΔH*_relax_ indicates that no rejuvenation occurs for the sample prepared at high casting temperature (HT samples).

**Fig. 2:**
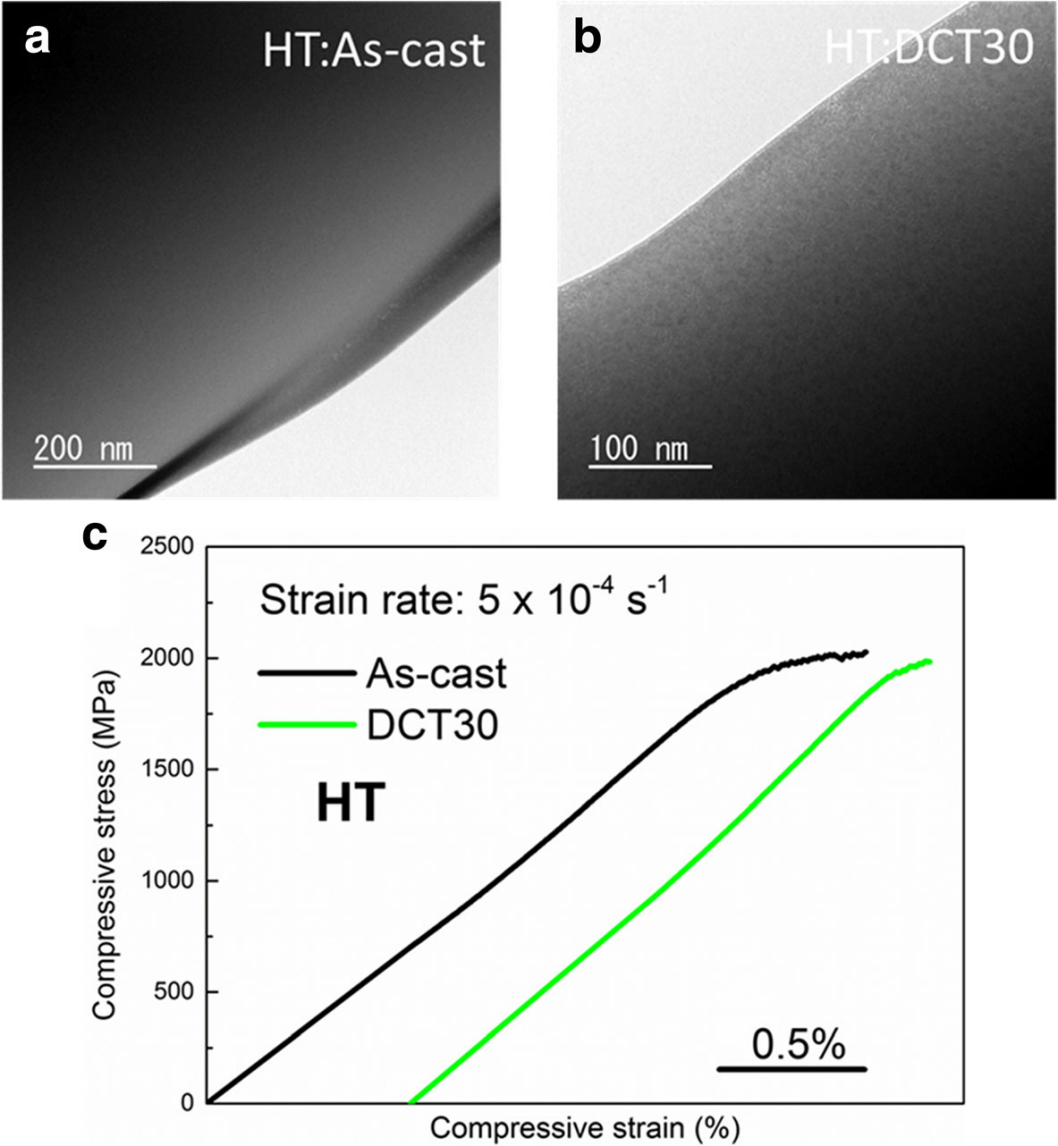
**a**, **b** Bright-field TEM images of As-cast and DCT30 samples cast at HT. **c** Compressive stress-strain curves of As-cast and DCT30 samples cast at HT

Figure 2a, b shows the bright-field TEM images of both As-cast and DCT30, respectively, which exhibits similar homogeneous maze-like amorphous structure of both samples without any crystalline phases. Figure 2c shows compressive stress-strain curves of both As-cast and DCT30 samples. No plasticization behavior is observed after DCT, the fracture strength and plastic strain for both samples are about 2000 MPa and 0.3%, respectively. The detailed data of compression test are summarized in Table 1.

**Table 1:** A summary of thermal and mechanical data for the sample in this work

		*T*_*g*_ (K)	*T*_*x*_ (K)	*t*_*x*_ (min)	*ΔH*_relax_ (J·g^−1^)	*E* (GPa)	*σ*_*f*_ (MPa)	*σ*_*y*_ (MPa)	*ε*_*f*_ (%)	*ε*_*p*_ (%)
HT	As-cast	690 ± 5	780 ± 4	12.6 ± 0.2	12.6 ± 0.5	105 ± 5	2027 ± 15	1865 ± 22	2.3 ± 0.2	0.5 ± 0.2
	DCT30	688 ± 4	781 ± 5	12.5 ± 0.3	12.9 ± 0.3	114 ± 4	1983 ± 20	1873 ± 15	1.9 ± 0.1	0.2 ± 0.1
LT	As-cast	695 ± 5	781 ± 3	13.9 ± 0.5	10.7 ± 0.2	123 ± 5	1998 ± 18	1765 ± 18	4.3 ± 0.2	2.8 ± 0.2
	DCT30	697 ± 3	774 ± 5	14.2 ± 0.3	13.0 ± 0.3	107 ± 6	2049 ± 19	1640 ± 13	5.9 ± 0.3	4.3 ± 0.3

*T*_*g*_ glass transition temperature, *T*_*x*_ onset crystallization temperature, *t*_*x*_ incubation time of crystallization, *ΔH*_relax_ enthalpy of relaxation, *E* Young’s modulus, *σ*_*f*_ fracture stress, *σ*_*y*_ yielding stress, *ε*_*f*_ fracture strain, *ε*_*p*_ plastic strain

Our previous study on the rejuvenation behavior of Zr_55_Cu_30_Al_10_Ni_5_ (at.%) BMG upon DCT has shown that the intrinsic core-shell heterogeneity is the main reason of rejuvenation during cyclically cooling and heating. The different elastic modulus of core and shell generates internal stress upon DCT, which causes the evolution of core region with more induced free volume [11]. Many researches have shown that the intrinsic heterogeneity of amorphous phase is related with the glass forming ability (GFA) of the alloy system [16, 17]. The BMG with a higher GFA possesses a more heterogeneous microstructure and subsequently causes rejuvenation upon DCT. However, for the sample in the present study, Zr_50_Cu_40_Al_10_ (at.%), the GFA is not as high as Zr_55_Cu_30_Al_10_Ni_5_ (at.%) [18, 19], thus, the more homogenous microstructure of Zr_50_Cu_40_Al_10_ cannot generate effective internal stress to rejuvenate the sample upon DCT.

### Rejuvenation Behavior of LT Samples

**Fig. 3:**
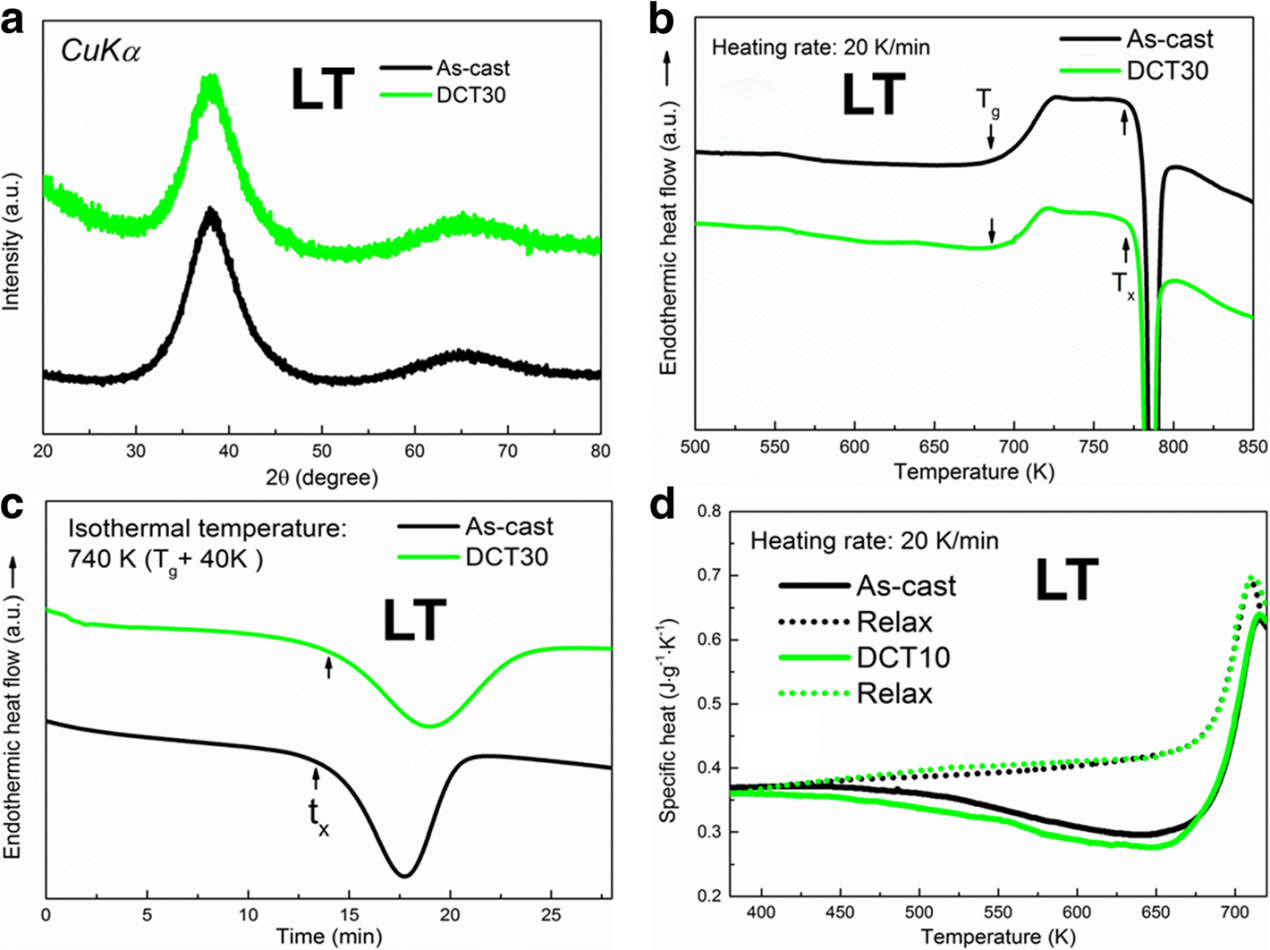
**a** XRD and **b** DSC curves of both As-cast and DCT30 samples cast at LT. **c** Heat-flow as a function of time during isothermal annealing (740 K) and **d** specific heat of both As-cast and DCT30 samples cast at LT

Figure 3a shows the XRD patterns of both As-cast and DCT30 for LT samples, which are cast from a lower casting temperature (LT). Similar to HT samples, only a broad peak without any crystalline peaks is detected for each sample. The *T*_*g*_ and *T*_*x*_ are also very close, as shown in Fig. 3b. However, the incubation time of crystallization for DCT30 is longer than that of As-cast sample (Fig. 3c), which is different from HT samples. Furthermore, the enthalpy of relaxation for both samples, which are calculated based on the data from Fig. 3d, shows a higher value of DCT30 than As-cast. The detailed data of thermal properties are summarized in Table 1.

Previous study has shown that once the BMGs are rejuvenated, the density decreases because of more induced free volume. The densities of both As-cast and DCT30 for HT and LT samples are measured, 6.930 ± 0.004 g/cm^3^ (As-cast) and 6.929 ± 0.004 g/cm^3^ (DCT30) for HT samples and 6.957 ± 0.004 g/cm^3^ (As-cast) and 6.931 ± 0.010 g/cm^3^ (DCT30) for LT samples. The reduced free volume (*x*) can be calculated based on densities [11, 12]:

2 $$ x=\frac{v_f}{\gamma {v}^{\ast }}=\frac{2\left({\rho}_c-\rho \right)}{\rho }, $$

where *v*_*f*_ is the average free volume per atom, *γ* is the correction term for the free volume overlap, v^*^ is the critical value of free volume for atomic diffusion, *ρ* is the density of the sample, and *ρ*_*c*_ is the density of a sufficiently crystallized sample, herein measured to be 6.971 ± 0.002 g/cm^3^ (annealed at 923 K for 3 h). Thus, *x* for HT samples can be calculated with Eq. (2), 1.18% for As-cast and 1.21% for DCT30. The similar value indicates that no more free volume has been induced upon DCT and no rejuvenation occurs for HT samples. For LT samples, the densities include both amorphous phase and nano-clusters. However, the calculation of *x* should base on the density of monolithic amorphous phase. Thus, we further calculate the density of amorphous phase in LT samples by using the rule of mixture as follows [20]:

3 $$ \rho ={\rho}_a{V}_a+{\rho}_{nc}{V}_{nc}, $$

where *ρ* is the total density, and *ρ*_*a*_ and *ρ*_*nc*_ are the densities of the glassy phase and nano-clusters, respectively. *V*_*a*_ and *V*_*nc*_ are the volume fractions of the glassy phase and nano-clusters, respectively. To calculate *ρ*_*a*_, the volume fraction of nano-clusters (*V*_*nc*_) should be clarified. To evaluate the *V*_*nc*_, we measured the crystallization enthalpy (*ΔH*_*s*_) by DSC from Fig. 3b (the area of exothermic crystallization peak). Thus, *V*_*nc*_ can be calculated as [21] follows:

4 $$ {V}_{nc}=1-\frac{{\Delta  H}_s}{{\Delta  H}_r}, $$

where *ΔH*_*r*_ is the crystallization enthalpy of the fully amorphous state and here we used the data of As-cast of HT sample (44.5 J/g). *ΔH*_*s*_ of As-cast and DCT30 are 41.0 and 40.7 J/g, respectively. Thus, *V*_*nc*_ are calculated to be 7.8% and 8.5% for As-cast and DCT30, respectively. The similar *V*_*nc*_ before and after DCT indicates that the nano-clusters are stable and maintain no change upon DCT. In addition, the nano-clusters in LT samples may be B2-CuZr phase and thus *ρ*_*nc*_ is about 7.45 g/cm^3^ [22, 23]. By using the data shown above with Eqs. (2) and (3), *x* of As-cast and DCT30 are calculated to be 1.30% and 2.06%, respectively, which suggests that more free volume has been induced for LT samples upon DCT and the BMGs are rejuvenated. It agrees well with the results from thermal analysis.

**Fig. 4:**
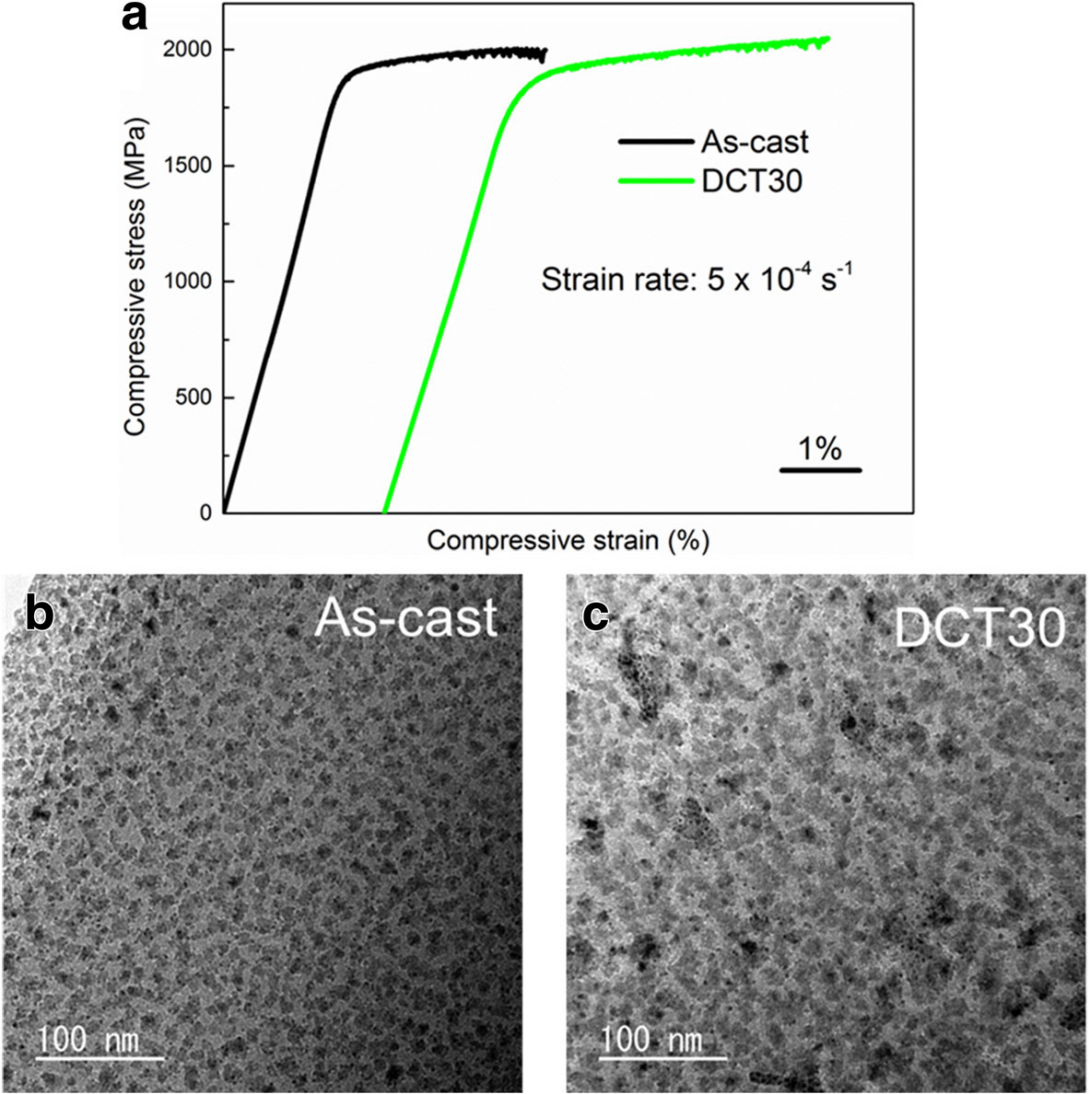
**a** Compressive stress-strain curves of As-cast and DCT30 samples cast at LT. **b**, **c** Bright-field TEM images of As-cast and DCT30 samples cast at LT

These results suggest that unlike HT samples, LT samples can be rejuvenated upon DCT. Figure 4a shows the compressive stress-strain curves of both As-cast and DCT30 samples which are fabricated at a low casting temperature (LT). Firstly, unlike HT As-cast sample, the LT As-cast sample shows obvious yielding and plasticity, which fractures at about 2000 MPa with 2.8% plastic strain. Furthermore, the DCT samples show better mechanical properties than As-cast samples, including higher fracture strength (~ 2050 MPa) and larger plastic strain (~ 4.3%). The rejuvenated state of DCT30 contributes to the improvement of plasticity, which induces more free volume and subsequently more shear transformation zone (shear bands) are activated or formed to accommodate the overall deformation [24]. The detailed data of compression test are summarized in Table 1.

The homogenous amorphous structure in HT samples cannot generate internal stress to rejuvenate themselves. In the contrary, the LT samples which have the same composition and cooling rate (same size of sample) can be rejuvenated upon DCT. This difference should originate from the microstructure. Figure 4b, c shows the TEM images of As-cast and DCT30 which are cast at low temperature, respectively. Apparently, very fine nano-sized clusters can be observed for both samples, which is different from the structure of HT sample shown in Fig. 2a, b.

**Fig. 5:**
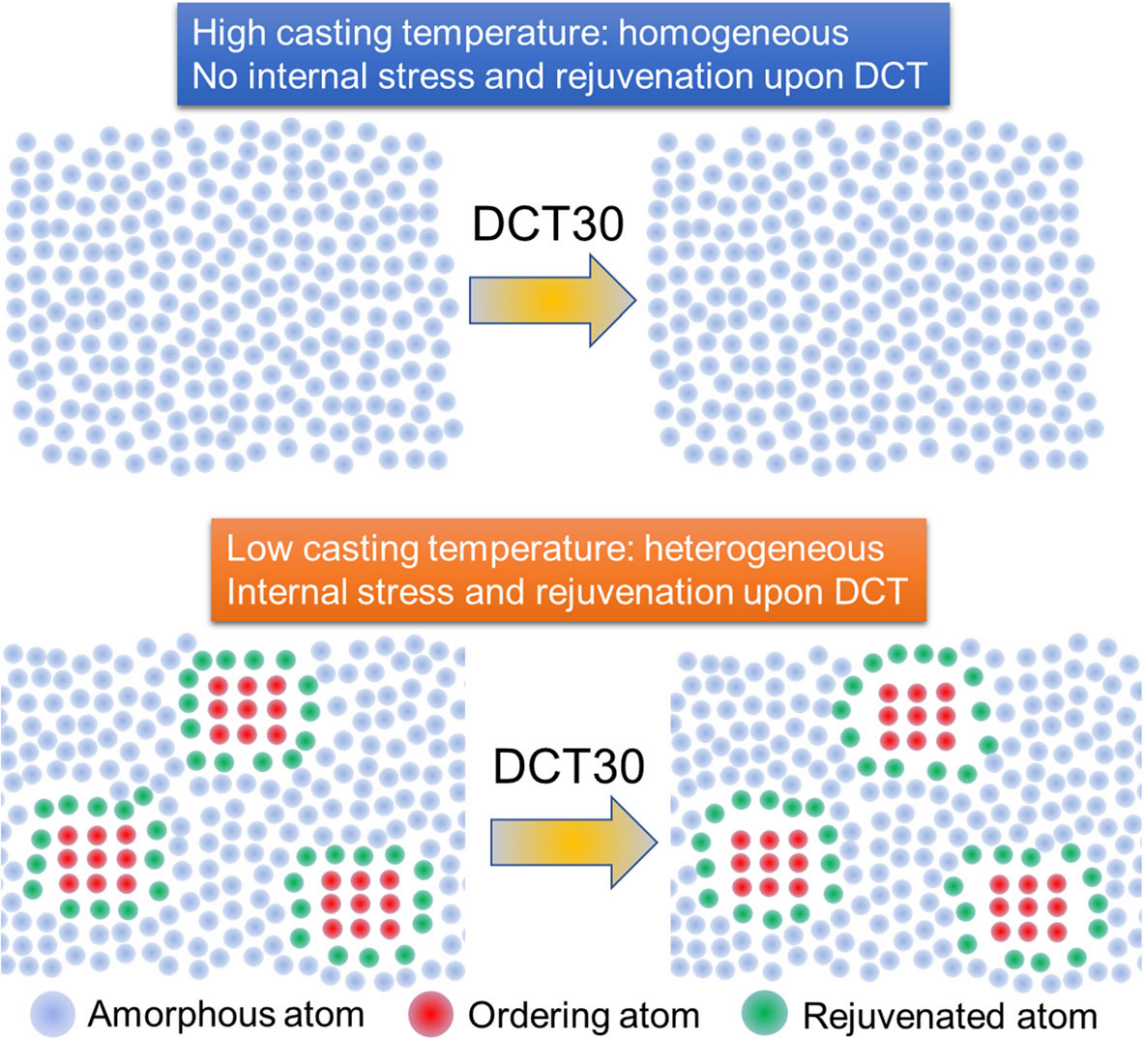
Schematic illustration of rejuvenation behavior for both HT and LT samples. Homogeneous structure of HT sample cannot generate internal stress upon DCT, while heterogeneity in LT samples helps generate internal stress at the interfaces. Therefore, rejuvenation behavior can be only observed in LT samples

Figure 5 shows the schematic illustration of rejuvenation behavior for both HT and LT samples. The HT sample possesses a quite homogeneous amorphous phase, thus no internal stress is generated upon DCT and therefore no rejuvenation occurs for HT samples. In the contrast, the nano-sized heterogeneity in LT samples should help generate the internal stress upon DCT because of the different intrinsic properties between two phases. Finally, LT samples can be rejuvenated. The internal stress (*σ*_*α*_) can be calculated as [25] follows:

5 $$ {\sigma}_{\alpha }=\Delta  \alpha \Delta  T\frac{2{E}_c{E}_a}{\left(1+{v}_a\right){E}_c+2\left(1-2{v}_c\right){E}_a}, $$

where *Δα* is the thermal expansion coefficient differences between the amorphous and crystalline phases, *ΔT* is the temperature change, *E*_*c*_ and *E*_*a*_ are the elastic modulus for the crystalline and amorphous phases, respectively, and *ν*_*c*_ and *ν*_*a*_ are the Poisson’s ratio for crystalline and amorphous phases, respectively. Previous study has shown that the nano-clusters may be B2-CuZr phase [22]. The thermal expansion coefficients for the amorphous and crystalline phases have been reported to be ~ 1.3 × 10^−5^ K^−1^ and 1.14 × 10^−5^ K^−1^, respectively [26], *E*_*c*_ and *E*_*a*_ have been reported to be ~ 77 and 123 GPa, respectively [27], and *ν*_*c*_ and *ν*_*a*_ have been reported to be ~ 0.385 and 0.383, respectively [28, 29]. *ΔT* was ~ 180 K (293 K to 113 K). Thus, by using Eq. (5), *σ*_*α*_ is calculated to be ~ 34 MPa, which causes local atomic rearrangement and also helps to rejuvenate the amorphous phase.

As the intrinsic heterogeneity of BMGS can affect the rejuvenation behavior of BMGs upon following thermal treatment, the reason why different casting temperatures can tailor the microstructures should be clarified. Zhu et al. have also found that the casting temperature can tailor the structure from fully amorphous state (at high casting temperature) to composite structure (at low casting temperature) [30]. When the metallic liquid is quenched from high temperature, the element in the liquid can be fully mixed and makes the liquid more homogenous. Thus, fully amorphous phase can be obtained. However, if the casting temperature is low, the element segregation can occur in very local area among the liquid, which are retained during the solidification. This segregation is considered to be the nuclei for the nano-clusters in LT samples. Furthermore, if the casting temperature is very low, we cannot produce amorphous samples even with high cooling rate. Therefore, varying the casting temperature can induce nano-sized heterogeneity in the amorphous matrix, which generates internal stress and rejuvenation during DCT.

## Conclusions

In the present study, the rejuvenation behaviors of Zr_50_Cu_40_Al_10_ (at.%) BMGs upon DCT have been investigated. At high casting temperature, for the fully mixing of elements, fully amorphous phase with quite homogeneous structure can be fabricated after quenching. No rejuvenation occurs for these samples because of the lack of internal stress during cyclically cooling and heating. In the contrary, at low casting temperature, for the element segregation, nano-cluster dispersed amorphous structure can be observed, which generates high internal stress and causes the rejuvenation of samples upon DCT. The rejuvenated sample with more free volume shows better plasticity than As-cast ones. These findings provide a novel method to tailor the microstructure of as-cast BMG samples, which affects both the mechanical properties and rejuvenation behavior during the following DCT treatment.

## Abbreviations

BMG: Bulk metallic glass
DCT: Deep cryogenic cycling treatment
DCT30: Thermal treated with 30 cycles
DSC: Differential scanning calorimeter
GFA: Glass forming ability
HT: High casting temperature
LT: Low casting temperature
TEM: Transmission electron microscope
XRD: X-ray diffraction
